# An integrated bioinformatic analysis of bulk and single-cell sequencing clarifies immune microenvironment and metabolic profiles of lung adenocarcinoma to predict immunotherapy efficacy

**DOI:** 10.3389/fcell.2023.1163314

**Published:** 2023-04-05

**Authors:** Mengling Li, Baosen Zhou, Chang Zheng

**Affiliations:** Department of Clinical Epidemiology and Center of Evidence-Based Medicine, The First Hospital of China Medical University, Shenyang, China

**Keywords:** lung adenocarcinoma, immunotherapy, metabolism, epithelia-mesenchymal transition, cancer-associated fibroblasts, single-cell multi-omics

## Abstract

Targeting the tumor microenvironment is increasingly recognized as an effective treatment of advanced lung adenocarcinoma (LUAD). However, few studies have addressed the efficacy of immunotherapy for LUAD. Here, a novel method for predicting immunotherapy efficacy has been proposed, which combines single-cell and bulk sequencing to characterize the immune microenvironment and metabolic profile of LUAD. TCGA bulk dataset was used to cluster two immune subtypes: C1 with “cold” tumor characteristics and C2 with “hot” tumor characteristics, with different prognosis. The Scissor algorithm, which is based on these two immune subtypes, identified GSE131907 single cell dataset into two groups of epithelial cells, labeled as Scissor_C1 and Scissor_C2. The enrichment revealed that Scissor_C1 was characterized by hypoxia, and a hypoxic microenvironment is a potential inducing factor for tumor invasion, metastasis, and immune therapy non-response. Furthermore, single cell analysis was performed to investigate the molecular mechanism of hypoxic microenvironment-induced invasion, metastasis, and immune therapy non-response in LUAD. Notably, Scissor_C1 cells significantly interacted with T cells and cancer-associated fibroblasts (CAF), and exhibited epithelial–mesenchymal transition and immunosuppressive features. CellChat analysis revealed that a hypoxic microenvironment in Scissor_C1elevated TGFβ signaling and induced ANGPTL4 and SEMA3C secretion. Interaction with endothelial cells with ANGPTL4, which increases vascular permeability and achieves distant metastasis across the vascular endothelium. Additionally, interaction of tumor-associated macrophages (TAM) and Scissor_C1 *via* the EREG/EFGR pathway induces tyrosine kinase inhibitor drug-resistance in patients with LAUD. Thereafter, a subgroup of CAF cells that exhibited same features as those of Scissor_C1 that exert immunosuppressive functions in the tumor microenvironment were identified. Moreover, the key genes (*EPHB2* and *COL1A1*) in the Scissor_C1 gene network were explored and their expressions were verified using immunohistochemistry. Finally, the metabolism dysfunction in cells crosstalk was determined, which is characterized by glutamine secretion by TAM and uptake by Scissor_C1 *via* SLC38A2 transporter, which may induce glutamine addiction in LUAD cells. Overall, single-cell sequencing clarifies how the tumor microenvironment affects immunotherapy efficacy *via* molecular mechanisms and biological processes, whereas bulk sequencing explains immunotherapy efficacy based on clinical information.

## 1 Introduction

Lung cancer is one of the most frequently diagnosed malignancies, with high recurrence and mortality. Lung cancers can be classified into two main types: the more common non-small cell lung cancer (NSCLC) and small cell lung cancer. Lung adenocarcinoma (LUAD) is the most prevalent NSCLC (Riihimäki et al., 2014). Most research on lung cancer progression and metastasis focuses on exploring cancer tissues and cells. However, the tumor microenvironment plays an equally important role in cancer progression and metastasis. Notably, treatment of metastatic NSCLC has greatly advanced through application of immune checkpoint blockers (ICB) of programmed cell death protein 1 (PD-1) and cytotoxic T lymphocyte-associated protein (CTLA-4) (Chae et al., 2018). Thus, identifying unique tumor microenvironments in advanced cancers will reveal mechanisms related to tumor-induced changes that can be potential targets for immunotherapy (Binnewies et al., 2018).

Different immune subtypes correlate with immunotherapy efficacy. Immunoinflammatory types exhibiting enrichment of T-cells and virulent T-cells are more likely to benefit from immunotherapy such as ICB. In contrast, immune-rejection types with lower T-cell infiltration, oncogenic pathway activation, aberrant neovascularization, and mesenchymal features are typically resistant. A comprehensive clinical analysis of interactions between tumor-infiltrating immune cells and tumor cells can predict immunotherapy efficiency (Zhang et al., 2022a). However, whether LUAD cell clusters can drive immune evasion and generate specific immune profiles associated with ICB ineffectiveness remains unclear (Messina et al., 2019). Currently, immunophenotyping of the tumor microenvironment is largely based on bulk sequencing (Ren et al., 2021). We propose that combining bulk sequencing with single-cell sequencing can resolve immune escape or activation features through the additional information on cell population interactions and molecular pathways (Ding et al., 2020). Consequently, we can better predict immunotherapy efficacy.

In this study, we applied the Scissor algorithm (Sun et al., 2022) to TCGA-LUAD and GEO samples and identified 2 cell groups most associated with immune subtypes of LUAD: Scissor_C1 and Scissor_C2. Our aim was to explore the crosstalk among these two groups of cancer cells, cancer associated fibroblast (CAFs), endothelial, and myeloid cells to elucidate the clinical significance and effects on ICB immunotherapy.

## 2 Materials and methods

### 2.1 Datasets

This study employed data from publicly accessible databases: TCGA-LUAD expression matrix compiled with UCSC Xena, LUAD-treated single-cell dataset, and GEO bulk datasets, GSE131907 and GSE68465. Clinical information and metadata of patients were obtained from the original publications. Immune efficacy analyses were performed using IMvigor210 (Necchi et al., 2017) and GSE78220 immunotherapy data.

For the GSE131907 dataset (Kim et al., 2020), epithelial cells and CAFs were extracted from cancer samples for subsequent analysis. For the GSE68465 and TCGA datasets, gene expression values were converted to TPM for subsequent analysis.

### 2.2 Identification of immune subtypes

Non-negative Matrix Factorization (NMF) clustering (optimal k value = 2) was performed to identify immune subtypes in TCGA-LUAD patients based on 297 immune-microenvironment signature genes (Bagaev et al., 2021). Next, gene set enrichment analysis (GSEA) was performed on identified immune subtypes to explore their immune activity. We applied ssGSEA to determine immune-related pathways of the subtypes; relative abundance of immune cells in each subtype was analyzed with the Cibersortx algorithm (Newman et al., 2019). Immunotherapy response scores were predicted with the EaSIeR package (Lapuente-Santana et al., 2021). This package uses bulk sequencing to assess patient response based on tumor cell infiltration, intracellular signaling, TF activity, and strength of intercellular communication. It has more dimensions for estimation than the TIDE (Jiang et al., 2018) score based on T-cell dysfunction and immunosuppressive T-cell rejection characteristics. Immunotherapy response was also evaluated with PD-L1 expression.

### 2.3 Single cell RNA-seq data processing

Three quality measures were applied to each epithelial cell: mitochondrial genes (≤20%), unique identifiers (UMIs), and gene counts (ranging from 100 to 150,000 and 200 to 10,000). The FindVariableFeatures function in the Seurat package identified 2,000 highly variable genes for RunPCA and UMAP random neighborhood embedding. Re-dimensional clustering and annotation of myeloid cells was performed with the Harmony algorithm (resolution = 0.3). Myeloid cell samples were integrated for UMAP descending clustering, and cell type annotations were identified *via* a literature search (Cheng et al., 2021a). The FindMarker function of Seurat was used to detect differentially expressed genes in various subpopulations.

### 2.4 Scissor algorithm for identifying phenotypically related cells

Bulk sequencing from TCGA-LUAD patients was correlated with single-cell data using the Scissor algorithm (Sun et al., 2022). The TCGA-LUAD immunophenotype was selected as the phenotype for logistic regression, and Scissors were run for each patient. The parameter alpha was set at 0.4 to filter out the most relevant immune subtypes.

### 2.5 Functional enrichment analysis

After Findmarker identified subgroups of differentially expressed genes, ClusterProfiler was used to analyze between-group differences. Functional analysis of subgroup marker genes was performed using Gene Ontology (GO), and GSEA was based on MsigDB data.

### 2.6 Cell communication analysis

CellPhoneDB 2 is a Python-based tool (Efremova et al., 2020) that analyzes intercellular communication at the molecular level. Scissors identified epithelial cells as the most relevant immune subtype, and thus, their communication with immune microenvironment-linked cells was determined using CellPhoneDB 2. Cell interaction pairs were further analyzed if the CellPhoneDB results returned a *p* < 0.05 and their ligands were TGFβ growth factors, CCL and CXCL families, or ECM-related encoded proteins. Interactions between immune subtype-associated Scissor cells and immune or stromal cells were explored using the CellChat package (Jin et al., 2021). This tool is based on cell interaction predictions from the 2021 CellChatDB database of experimentally validated ligand pairs.

### 2.7 Detecting metabolic modules and metabolomic changes in scRNA-seq

MEBOCOST (Zheng et al., 2022) was used to analyze metabolite communication between immune subtype-related Scissor cells and tumor-associated macrophages (TAM). This model estimates relative abundance of metabolites based on expression of genes encoding metabolic response enzymes. It also collects enzyme-related genes from the human metabolome (HMDB) and identifies cellular metabolite-sensor communication between cell populations.

We used scMetabolism (Wu et al., 2022) to analyze differences in metabolic pathway activity in single-cell datasets. scMetabolism is an R package based on quantifying metabolic activity at the single-cell level. It uses the VISION algorithm to score each cell and ultimately obtain the cell’s activity score in each metabolic pathway.

### 2.8 Gene regulatory network analysis

SCENIC (Aibar et al., 2017) is a new computational method for constructing regulatory networks and identifying different cellular states using scRNA-seq data. To measure differences in transcription factors or their target genes across Scissor epithelial cell clusters, pySCENIC analysis was implemented.

### 2.9 Data analyses

CIBERSORTx with default parameters was used to assess absolute abundance per cell type (half categorized into “high” and the other half into “low”). Kaplan-Meier analysis was performed to assess the prognostic value of cell clusters and evaluate their role in LUAD progression. The TCGA-LUAD and GSE68465 datasets were downloaded for survival analysis of the bulk RNA-seq cohort; their expression in TNM and pathological staging was also assessed. The IMvigor210 and GSE78220 datasets were included to determine the therapeutic effect of immunotherapy. All analyses were performed in R (version 4.10.0).

### 2.10 Drug sensitivity evaluation

Drug sensitivity of the TCGA-LUAD cohort was analyzed based on their TPM expression matrices using the R package “pRRophetic” (Geeleher et al., 2014).

### 2.11 Immunohistochemical validation

Immunohistochemical staining was performed on dewaxed and paracancerous LUAD tissue samples from the Department of Thoracic Surgery, The First Affiliated Hospital of China Medical University. After eliminating endogenous enzymes with a 3% peroxidase solution, tissues were incubated in 5% bovine serum albumin for 20 min at room temperature, then incubated with primary antibodies overnight. On the following day, tissues were incubated with the secondary antibody for 30 min and visualized with 3,3′-diaminobenzidine. Tissues were then dehydrated, sealed, and counterstained with hematoxylin. This study was approved by the Ethics Committee of the First Affiliated Hospital of China Medical University (NO.:AF-SOP-07-1.1-01), and conformed to the Declaration of Helsinki and Good Clinical Practice guidelines.

## 3 Results

**FIGURE 1:**
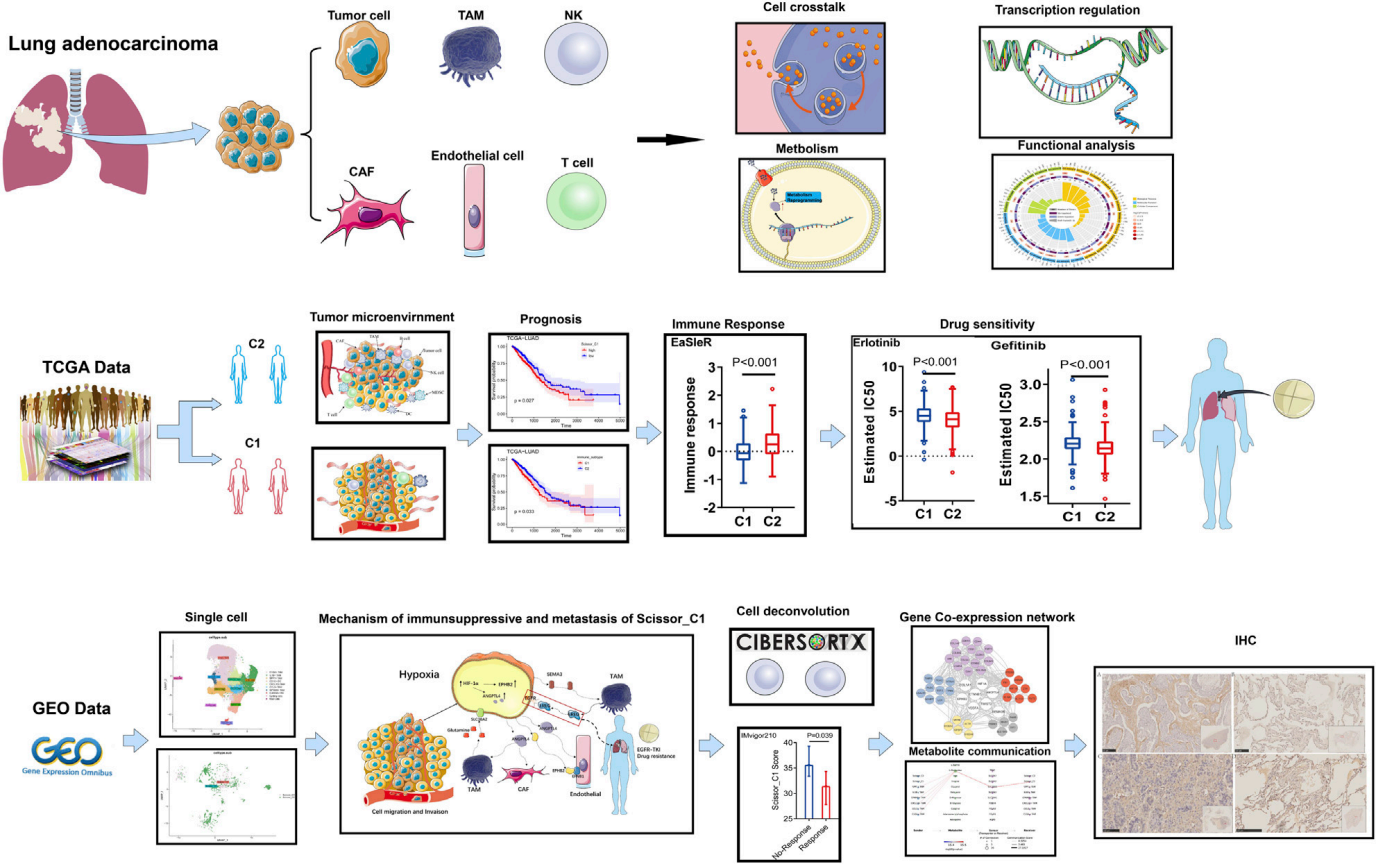
Overview of the work in this study.

The workflow diagram of the method used in this study is shown in Supplementary Figure S1. The outcome of NMF clustering on 297 immune microenvironment-related genes referred from the studies by Bagaev A (Bagaev et al., 2021). yielded 510 TCGA-LUAD patients. These individuals were grouped into two immune subtypes (cold and hot) for assessment of immune microenvironments based on bulk sequencing (Figure 1). Immunotherapeutic efficacy and Tyrosine kinase inhibitor (TKI) sensitivity of both immune subtypes were evaluated, and single-cell sequencing was applied to identify representative epithelial cells. Cell interaction analysis revealed that these epithelial cells differed significantly between subtypes in interactions with immune and stromal cells; metabolic mechanisms were then evaluated. Finally, SCENIC was used to construct the gene regulatory network related to the C1 subtype, and gene expression was verified using immunohistochemistry.

### 3.1 Identification of immune microenvironment heterogeneity in LUAD based on bulk dataset

**FIGURE 2:**
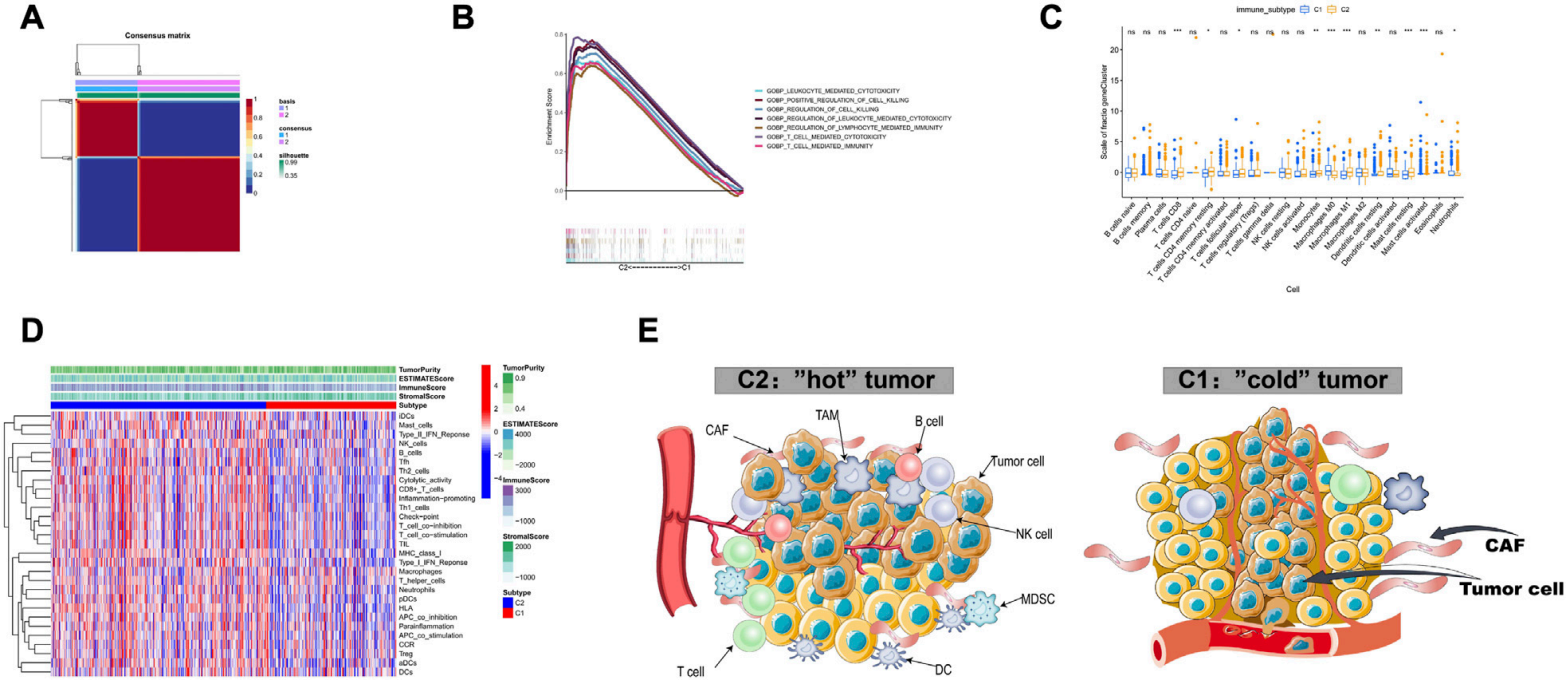
Identification of two immune subtypes based on TCGA-LUAD **(A)** Identification of two immune subtypes, C1 and C2, based on 510 samples with NMF optimal k = 2. **(B)** GSEA analysis showed that C2 had higher immune activity compared to C1. **(C)** Relative abundance of immune cells in both immune subtypes. **(D)** Heatmap of the 29 immunogenomes of the two immune subtypes, with higher cytotoxicity, immune checkpoint and pro-inflammatory process activity in C2 compared to C1. **(E)** Tumor microenvironment profiles of the two immune subtypes.

**FIGURE 3:**
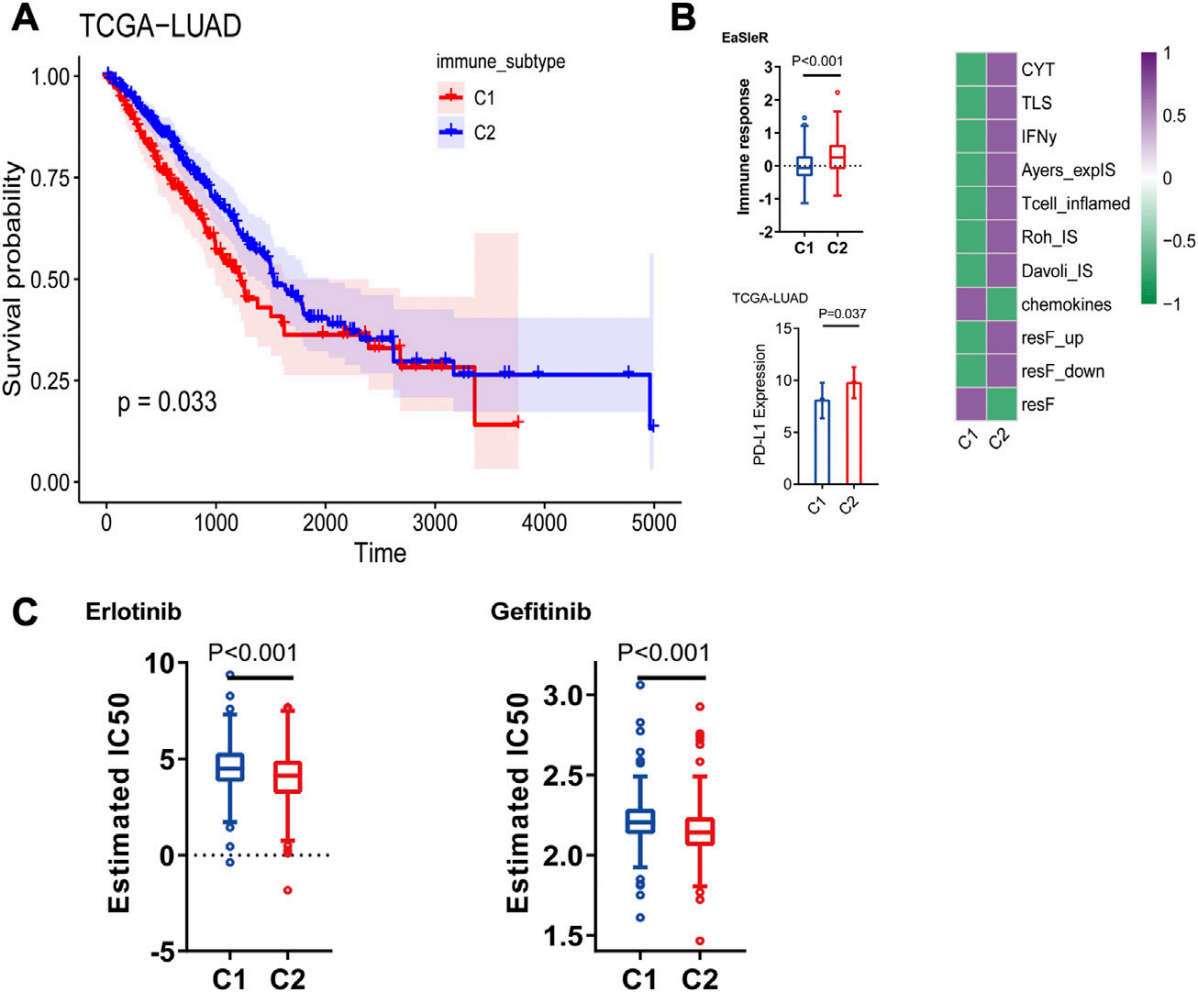
**(A)** Prognostic value of both immune subtypes, C1 subtype had poor prognosis compared to C2, and C1 immunotherapy response score was lower. **(B)** Immunotherapy efficacy (EaSIeR score and PD-L1 expression) **(C)** Resistance of two immune subtypes to EGFR-TKI drugs.

We classified TCGA-LUAD patients into two immune subtypes based on 297 tumor microenvironment-related genes (Supplementary Table S1). Of the two immune subtypes, C1 and C2 (Figure 2A), the latter was more immunoreactive than the former, according to GSEA (Figure 2B). First, CIBERSORT revealed inconsistent immune-cell content in the two subtypes, with C2 possessing greater immune cell infiltration (“hot” tumor characteristics), whereas C1 had significantly less immune cell infiltration (“cold”tumor characteristics) (Figures 2C, D). Additionally, the C1 and C2 subtypes were associated with poor and good prognoses, respectively (Figure 3A). Thereafter, immunotherapy response scores were derived using EaSIeR package, and drug sensitivity scores using pRRophetic package (Supplementary Table S2). The C1 subtype had significantly lower EaSIeR scores (*p* < 0.001) and PD-L1 expression (*p* = 0.037) than the C2 subtype (Figure 3B). Subsequent evaluation of TKI resistance in the two immune subtypes revealed that the IC50 of erlotinib and getfitib was significantly higher in C1 than in C2, and that C1 subtype was more likely to develop resistance to TKI drugs (Figure 3C). Thus, the C1 subtype is more likely to resist immunotherapy, whereas the C2 subtype is more likely to benefit from immunotherapy. These results show the stability of the TCGA immune classifier.

### 3.2 Integrated scRNA-seq analysis to dissect and cluster immune-related epithelial cell populations in LUAD

**FIGURE 4:**
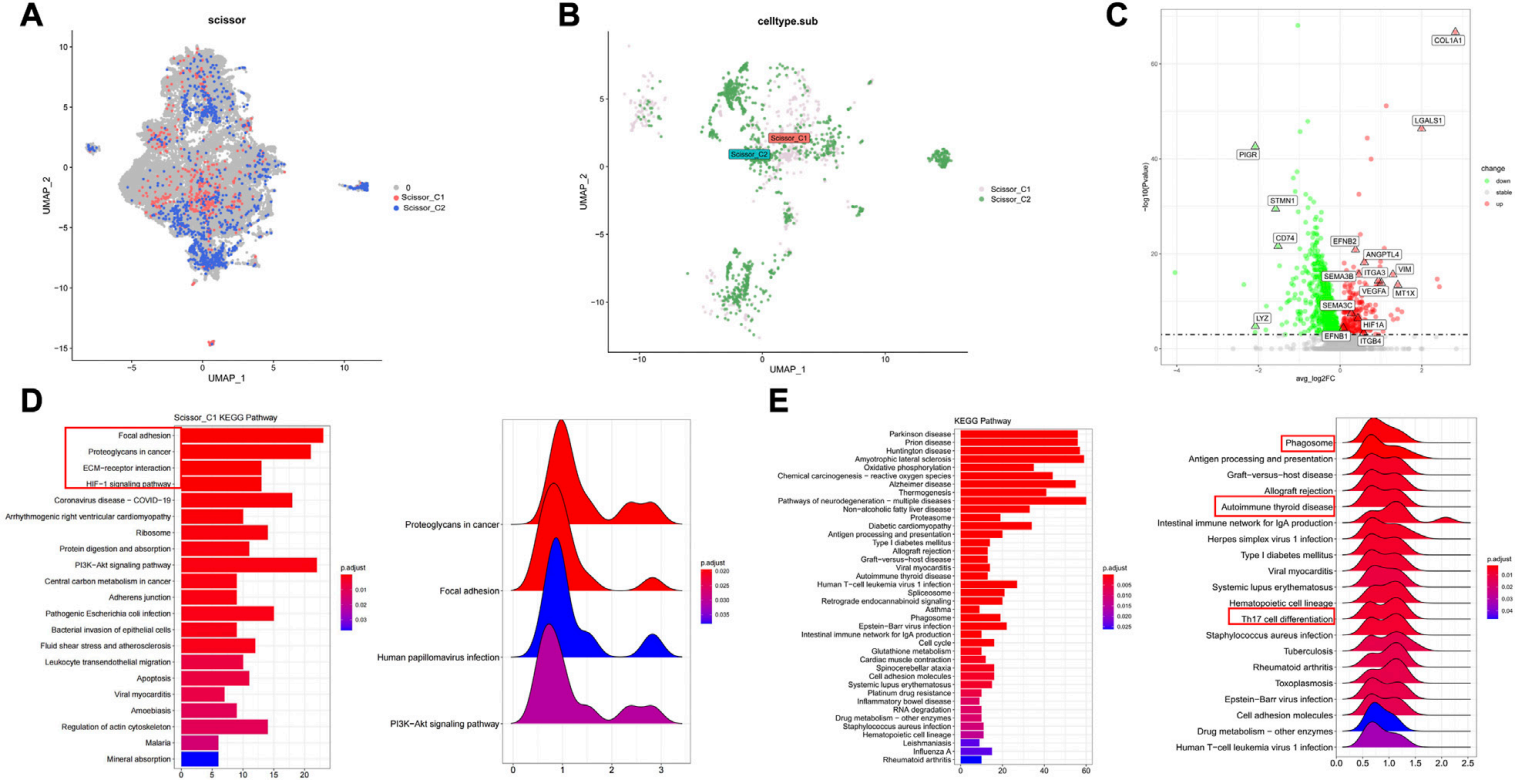
Characterization of two groups of Scissor epithelial cells **(A,B)** The Scissor algorithm identifies the two epithelial cells most associated with the two immune subtypes, Scissor_C1 and Scissor_C2 cells. **(C)** Scissor_C1 significantly up- and downregulated genes. **(D)** KEGG and GSEA enrichment analysis of Scissor_C1 revealing hypoxia and EMT related biological processes (Focal adhesion, ECM-receptor interaction and HIF-1 signaling pathway. **(E)** Revealing Scissor_C2 enrichment in oxidative phosphorylation and immune activity related pathways (antigen presentation, Th17 cell differention).

Malignant cells associated with immune subtypes were first identified in the scRNA-seq dataset, we subjected 36,071 epithelial cells from multiple tissue sections in GSE131907 dataset to gene filtering, normalization, and principal component analysis. Next, the original samples were visualized using UMAP plots (resolution = 0.7). The Scissor algorithm identified 562 and 1,082 epithelial cells most associated with the C1 and C2 subtypes, respectively (Figures 4A, B). The most relevant epithelial cells subgroups to the C1 subtype was labeled as Scissor_C1, and the most relevant epithelial cells subgroup to the C2 subtype was labeled as Scissor_C2.

Tumors containing epithelial cells with epithelial–mesenchymal transition (EMT) characteristics are more likely to metastasize. Scissor_C1 showed significant EMT and angiogenic characteristics, being significantly upregulated in the following genes: mesenchymal COL1A1, EMT-related VIM, angiogenesis-related VEGFA and EFNB1, hypoxia-related HIF1A, and metastasis-related ANGPTL4 (Figure 4C). Based on the above differential gene results, the biological functions of these two types of tumor cells were explored using KEGG and GSEA. The results of KEGG analysis and GSEA revealed that Scissor_C1 cells were significantly enriched in the adhesion spot kinase, extracellular matrix interaction, and hypoxia-related pathways (Figure 4D). Therefore, Scissor_C1 may undergo EMT leading to invasive metastasis, in close association with a hypoxic environment. Scissor_C2 cells were enriched in ROS- and oxidative phosphorylation-related pathways, as well as in phagosome- and antigen presentation-immune pathways (Figure 4E).

**FIGURE 5:**
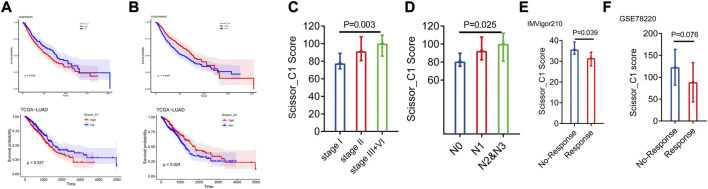
**(A,B)** Prognostic analysis of two groups of Scissor cells deconvoluted to bulk sequencing **(C,D)** Scissor_C1 cells exhibit different abundance in stage and N-stage deconvolution. **(E,F)** Scissor_C1 was highly expressed in the immunotherapy non-responsive group.

The reference matrix file of CIBERSORTx based on single-cell expression matrices of Scissor_C1 and Scissor_C2 was constructed to investigate the clinical role of the Scissor_C1 subgroup. Additionally, we calculated the absolute abundance of both Scissor cells in TCGA-LUAD and GSE68465 datasets. The results showed that high Scisssor_C1 abundance led to poor prognosis, whereas high Scissor_C2 abundance led to a good prognosis (Figures 5A, B). Scissor_C1 was highly expressed in stages III and IV and regional lymph node number one, indicating that these cells may be related to tumor progression (Figures 5C, D). Finally, high Scissor_C1 infiltration in immune non-responsive groups of IMvigror210 and GSEA78220 suggested that elevated C1 absolute abundance is related to immunotherapy non-response (Figures 5E, F), suggesting that Scissor_C1 may suppress immune response and develop malignant tumor metastasis in the hypoxic tumor immune microenvironment.

### 3.3 Cell communication network uncovers potential crosstalk among diversity of tumor cells and the TME point in LUAD

We used CellphoneDB to probe the interaction between scissor tumor cells and immune and stromal cells and showed that scissor tumor cells interacted significantly with T, CAF, endothelial, and Myeloid cells.

**FIGURE 6:**
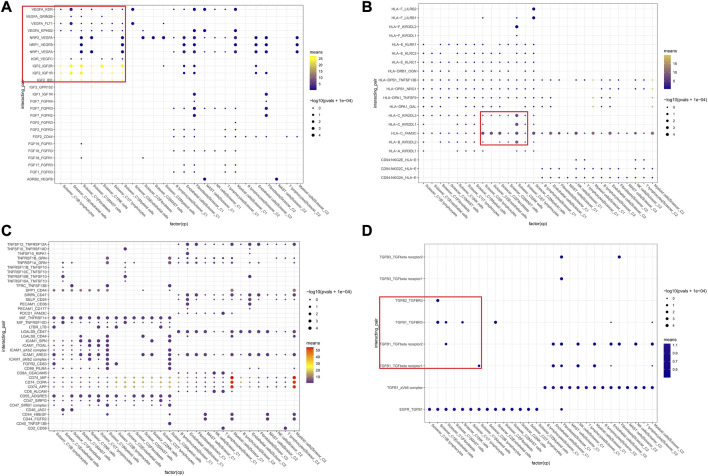
Cellular communication networks of two groups of Scissor cells **(A)** Communication between Scissor cells and immune, stromal cell growth factors. **(B)** Communication between Scissor cells and MHC-like molecules of immune, stromal cells. **(C)** Communication between Scissor cells and immune, stromal cell colony-stimulating factors **(D)** TGFβ analysis and cell growth factor communication.

Based on KEGG and GSEA results, we selected specific receptor–ligand pairs (e.g., growth factors, MHC-like molecules, CXCL family, and immune checks) for cellular interaction analysis. We found that Scissor_C1 cells expressed KDR (VEGFR-2) and FLT1 (VEGFR-1) receptors bind to VEGFA/VEGFB released from CAFs and endothelial cells (Figure 6A). This process stimulated tumor growth and metastasis, along with the elevated expression and secretion of growth factors in Scissor_C1 cells cluster. First, Insulin-like growth factor 2 (IGF2)-IGF1R/IGF2R was upregulated to interact with immune cells and CAFs. Second, upregulated TGFβ also interacted with CAF, endothelial, and myeloid cells, promoting the mesenchymal phenotype (Figure 6D). Hypoxia induced high expression of TGFβ in tumor cells, and TGFβ stimulated angiogenesis in the tumor vicinity. Simultaneously, TGFβ induced the expression of vascular endothelial growth factor (VEGF). Furthermore, TGFβ increased the expression of hypoxia-inducible factor (HIF) by inducing the pathway of high SMAD expression. Third, overexpression of TGFβI inhibited T and NK cell activity, causing immunosuppression (Yang et al., 2010). Owing to weak co-stimulation and tumor microenvironment signaling, TGFβI may reduce Satb1 induction, thereby increasing PDCD1 levels and further locking T-cell in a depleted state (Celada et al., 2018). This mechanism may allow potential immunotherapies to reverse depleted T-cell and enhance antitumor immune efficacy. Aberrant expression of FAM3C protein, encoded by interleukin-like EMT inducer (ILEI), in NSCLC tumors enhances cell transformation and stimulates distant lung tumor colonization, and a study on mice demonstrated that FAM3C is an oncogenic factor and a driver of distant metastasis in NSCLC (Thuya et al., 2023). The depleted T-cell in this study may interact with Scissor_C1 tumor cells through the PDCD1_FAM3C receptor ligand pathway, thereby promoting distant metastasis of LUAD (Figure 6C). Lastly, Scissor_C1 downregulated MHC class I to promote tumor immune escape and immunosuppression (Figure 6B). In contrast, Scissor_C2 upregulated MHC class I to interact with the tumor microenvironment, as well as with NK cells and macrophages, thus stimulating immune activation.

### 3.4 Lymphocytes divert towards immunosuppressive and differentiation phenotypes

**FIGURE 7:**
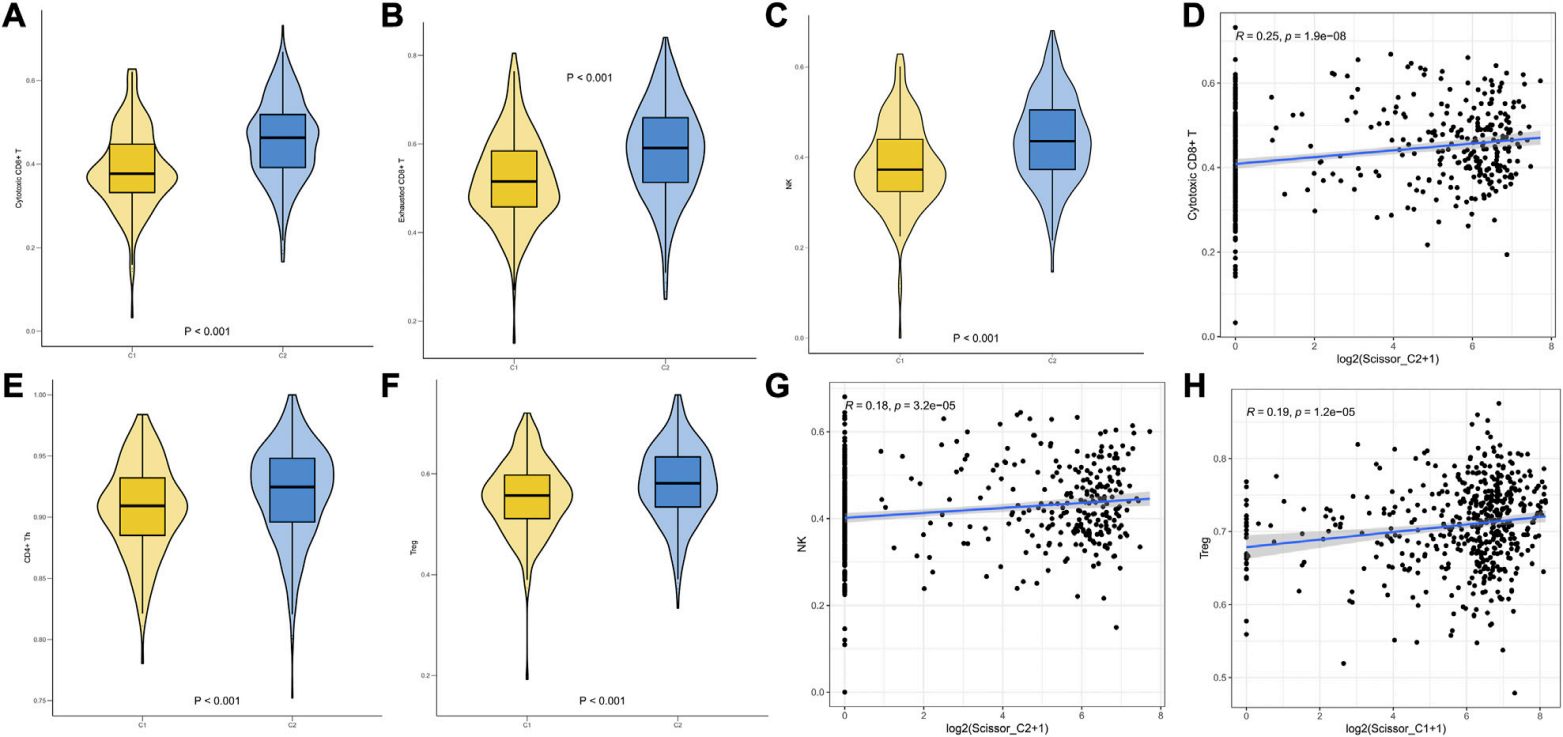
**(A–F)** ssGSEA infiltration scores of T-cells in two groups of immune subtypes. (**D,G,H**) Correlation analysis of Scissor cells with T-cells in both groups.

We performed ssGSEA on TCGA-LUAD data using infiltration scores calculated per patient for the first 25 characteristic markers of cytotoxic CD8^+^ T, Treg, NK, CD4^+^ Th, and exhausted cells in GSE131907. The results revealed that Cytotoxic CD8^+^ T and NK infiltration scores were significantly higher in C2 than in C1 (Figures 7A–C). We then demonstrated that Scissor_C2 abundance was positively correlated with Cytotoxic CD8^+^ T (r = 0.25, *p* = 1.9e-08) and NK cells (r = 0.18, *p* = 3.2e-05) (Figures 7D–G). Scissor_C1 was positively correlated with immunosuppressive Treg cells (r = 0.19, *p* = 1.2e-05) (Figure 7H).

### 3.5 Top ligand-receptor interactions between LUAD tumor cells and the TME point to mechanisms of angosisgene and immune evasion

**FIGURE 8:**
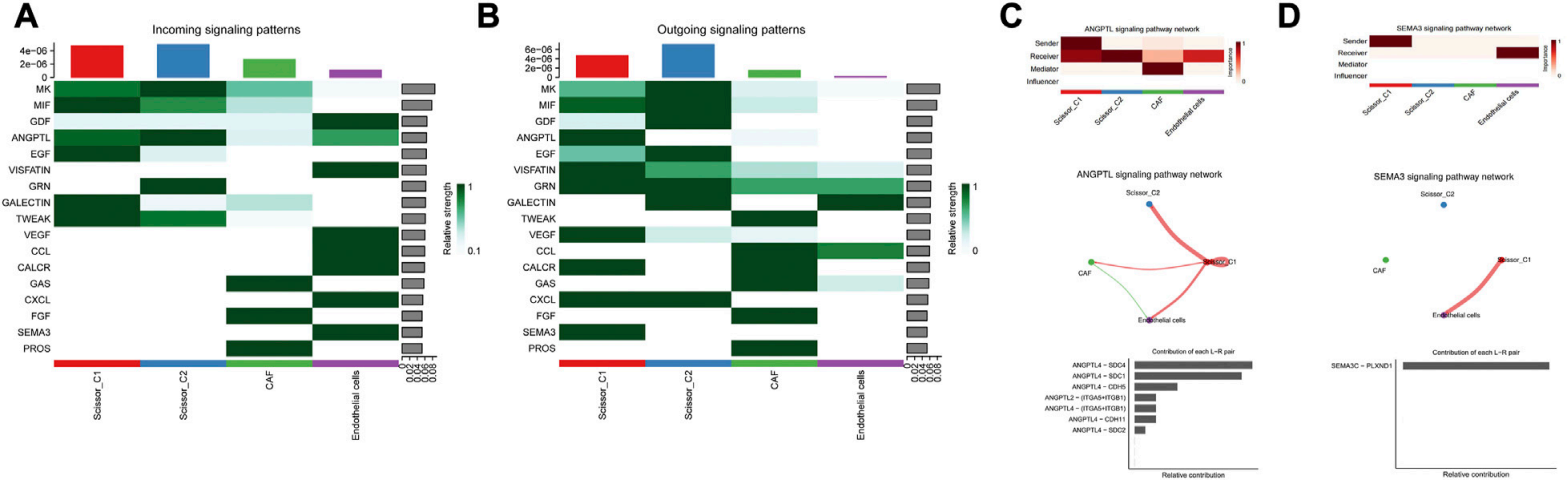
**(A)** Scissor and stromal cell signaling-related pathways. **(B)** Scissor and stromal cell release signaling-related pathways. **(C)** Scissor_C1 cells interact with CAF, endothelial cells through the release of ANGPTL4. **(D)** Scissor_C1 releases SEMA3C to interacts with endothelial cells.

CellChat was used to probe the crosstalk of scissor tumor cells and other cell types involved in molecular signaling pathways. Further CellChat analysis of these two immune-related ligand receptor pairs followed. For the putative ANGPTL4 signaling network, Scissor_C1 subpopulation was the sole source of action on CAF and endothelial cells. Scissor_C1 interacted significantly with CAF and endothelial cells, promoting cancer cell metastasis through ANGPTL4 secretion. The relative contribution of each ligand pair in the signaling pathway and the role of each cell population has been determined, with endothelial cells receiving the strongest signal. The hypoxic microenvironment contributed to ANGPTL4 secretion. The binding of ANGPTL4 to endothelial cells disrupted their connectivity, increased permeability of pulmonary capillaries, and promoted transvascular endothelial migration of tumor cells (Figures 8A–C). The activation of TGFβ signaling pathway in hypoxic microenvironment was shown to promote the release of ANGPTL4 from tumor cells as an important mechanism to promote angiogenesis. This suggests an idea for the receptor study of ANGPTL4.

For the crosstalk with the vascular endothelial cell signaling pathway, the receptors of ANGPTL4 in the cellular crosstalk have not been clarified, and in the present study, new ANGPTL4 receptors have been identified, and different ANGPTL4 receptors may have different functions including SDC family, CDH5, ITGB1, ITGA5, and CDH11 (Huang et al., 2011). The SEMA3C/PLXND1 ligand–receptor pair of Scissor_C1 acting on endothelial cells was uniquely directed and SEMA3C signaling protein played a role in vascular guidance (Figure 8D). It bound to a specific receptor PLXND1 (Yang et al., 2015), thereby activating a complex anti-angiogenic intracellular signaling cascade response. Moreover, SEMA3C/PLXND1 was shown to increase ANGPTL4/integrin receptor activation, further enhancing the angiogenic effect.

**FIGURE 9:**
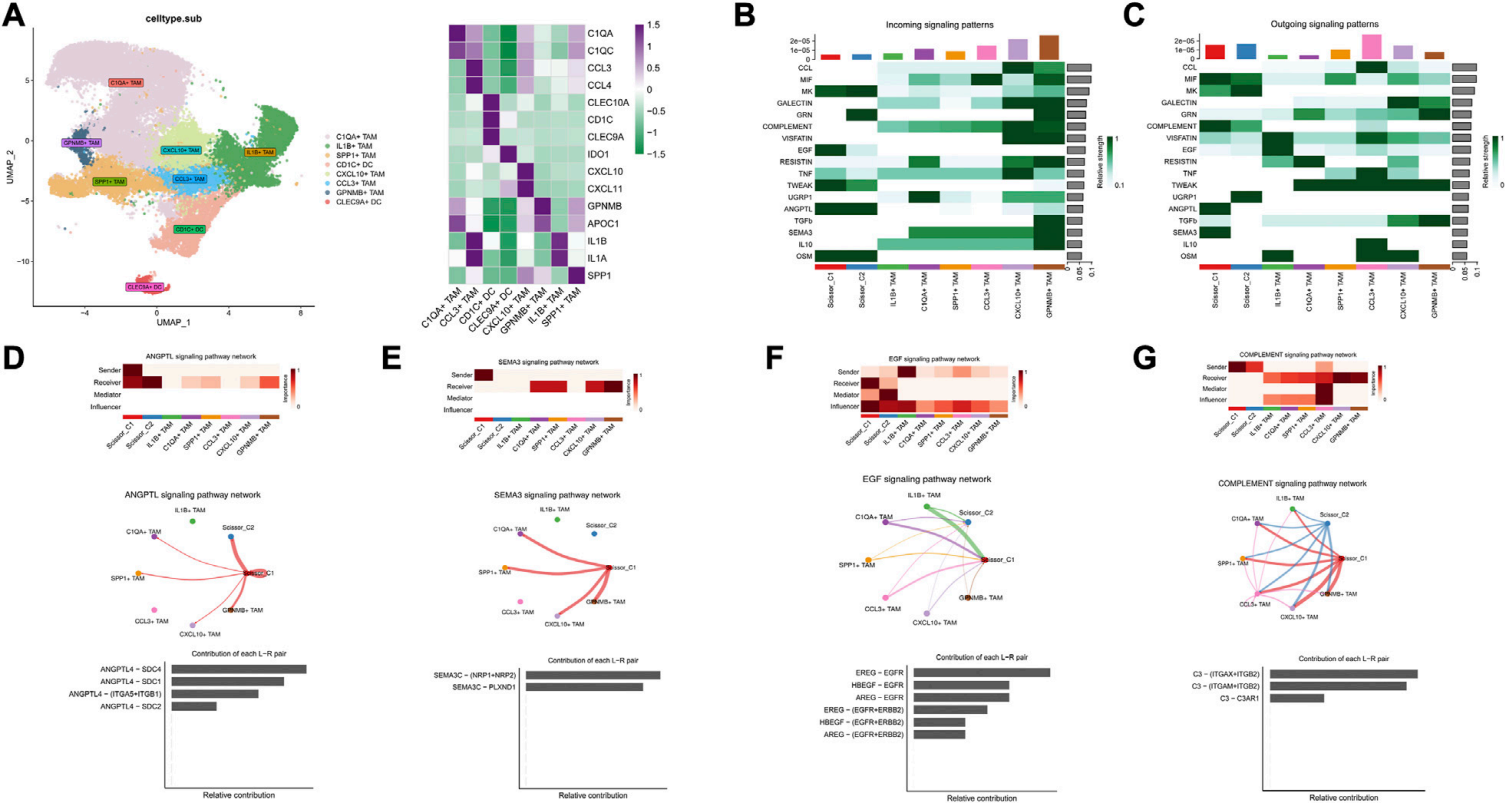
**(A)** Myeloid cell descending clustering and cell annotation, characteristic marker gene expression. **(B)** Signaling reception-related pathways in Scissor and TAM cells. **(C)** Scissor cell and TAM cell release signaling-related pathways. **(D)** Interaction of Scissor_ C1-released ANGPTL4 with TAM cells. **(E)** Scissor_C1 cells release SEMA3C to interact with TAM cells. **(F)** TAM interacts with Scissor_C1 *via* the EGF signaling pathway. **(G)** Scissor_C1 interacts with TAM cells through the complement pathway.

Based on marker annotation, myeloid cells were grouped into eight subtypes: C1QA + TAM, IL1B + TAM, SPP1+ TAM, CD1C + DC, CXCL10 + TAM, CCL3+ TAM, GPNMB + TAM, and CLEC9A + DC (Figure 9A). Crosstalk analysis with TAM cells showed that ANGPTL4 and SEMA3C signaling pathway also exhibited strong output signals in Scissor_C1 and were the only signaling sources interacting with C1QA + TAM, SPP1+ TAM, CXCL10 + TAM, and GPNNB + TAM, which exhibited M2-like anti-inflammatory macrophages (Figures 9B, C). CellChat analysis indicated that Scissor_C1 secreted SEMA3C to interact with NRP1/NRP2 receptor on TAMs (Figure 9E). SEMA3C induced polarization toward M2-like macrophages by upregulating NRP2 expression through activation of the Hedgehog pathway. Previous research showed that in hypoxic microenvironments, TWIST transcription factor interacts with NRPI to elevate SEMA3C expression (Yang et al., 2022), which promotes tumor cell angiogenesis and perineural invasion, suggesting that SEMA3C may mediate a key gene for immunosuppression under hypoxic microenvironment.

Additionally, in both Scissor_C1 and Scissor_C2 cell subsets, Scissor_C1 in particular was found to be the main receptor of the EGF signaling pathway, possibly dominated by IL1B + TAM, whereas Scissor_C2 exhibited a strong regulatory role. TAM highly expressed EREG to interact with EGFR receptors on Scissor_C1 (Figure 9F). The interaction of TAMs with Scissor_C1 through the EREG/EGFR pathway probably contributed to EGFR-TKI resistance in patients with NSCLC (Figure 3C).

### 3.6 CAFs divert towards immunosuppressive and differentiation phenotypes

**FIGURE 10:**
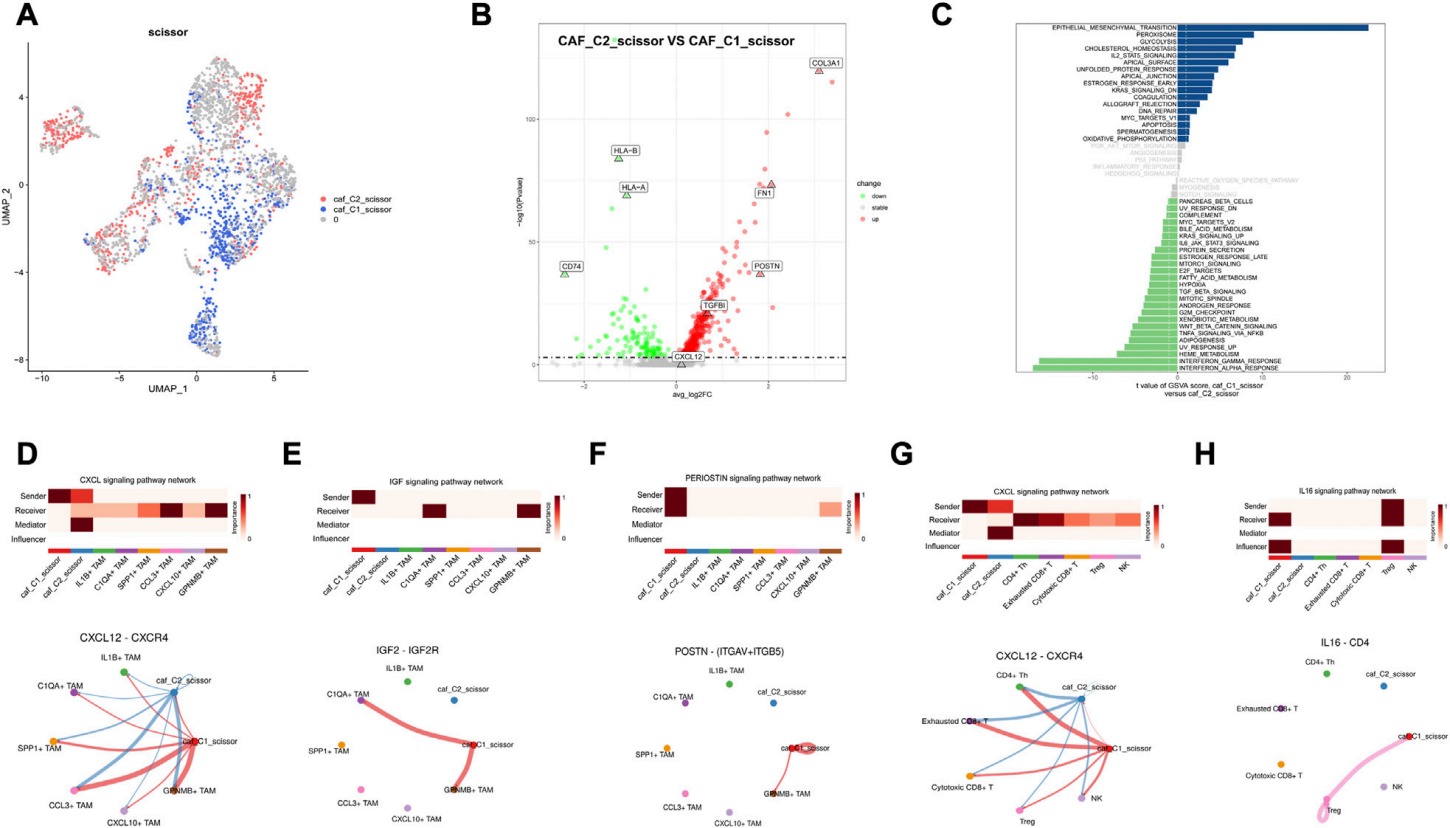
Results of Scissor identification in CAF cells **(A)** Visualization of UAMP in CAF cells, caf_C1_scissor for Scissor cells related to C1 subtype, and caf_C2_scissor for Scissor cells related to C2 subtype. **(B)** Volcano plot of differential expression of caf_C1_scissor and caf_C2_scissor. **(C)** Map of GSVA pathway in CAF-related Scissor cells. **(D,G)** caf_C1_scissor interacts significantly with TAM and T-cells *via* CXCL12_CXCR4. **(E)** caf_C1_scissor1 interacts significantly with C1QA + TAM *via* IGF signaling pathway. **(F)** caf_C1_scissor interacts significantly with TAM cells through the POSTN signaling pathway. **(H)** caf_C1_scissor interacts significantly with Treg cells *via* IL16 signaling pathway.

Previous findings suggest great heterogeneity in tumor TME and that CAF activation and classification in microenvironment have a great impact on immune cell infiltration and tumor metastasis; therefore, we further explored the homotypic and heterotypic between CAF immune subtypes by scissor algorithm. The most relevant CAF subgroup to the C1 subtype was labeled as caf_C1_scissor, and the most relevant CAF subgroup to the C2 subtype was labeled as caf_C2_scissor (Figure 10A). Differential expression analysis was performed to further understand the functions of these two groups of CAF cells. TGFBI, COL3A1, and CXCL12 were significantly upregulated in caf_C1_scissor, which mainly expressed genes characteristic of activated CAF, which secreted pro-inflammatory factors that enhanced EMT and stemness of tumor cells and contributed to tumor metastasis. CD74 and HLA-related genes are upregulated in caf_C2_scissor. caf_C2_scissor may be closer to the antigen-presenting CAF phenotype, which are similar to antigen-presenting CAFs and can activate CD4^+^ T-cell in an antigen-specific manner confirming their immunomodulatory capacity, similar in function to the C2 subtype of Scissor_C2 tumor cells (Figures 10B, C).

Combined with the analysis of cell communication results, caf_C1_scissor was found to be a CAF subgroup with immunosuppressive function. High expression of CXCL12 by caf_C1_scissor interacted significantly with TAM and T-cells (Figures 10D, G), and CXCL12 was an important ligand for CAF to achieve immunosuppression. Secretion of IGF2 by caf_C1_scissor interacted significantly with C1QA + TAM, whereas high expression of POSTN ligand interacted significantly with GPNMB + TAM (Figures 10E, F). The above demonstrates that caf_C1_scissor and Scissor_C1 have the same effect in preventing immune cell infiltration, attenuating immune cytotoxicity and promoting tumor invasion and metastasis. It shows that this study is robust in constructing immune subtypes, and it is reasonable to explore the cell subpopulations associated with immune subtypes using the scissor algorithm and to validate key ligand–receptor pairs.

### 3.7 Identification of key genes in Scissor_C1 cells and validation

**FIGURE 11:**
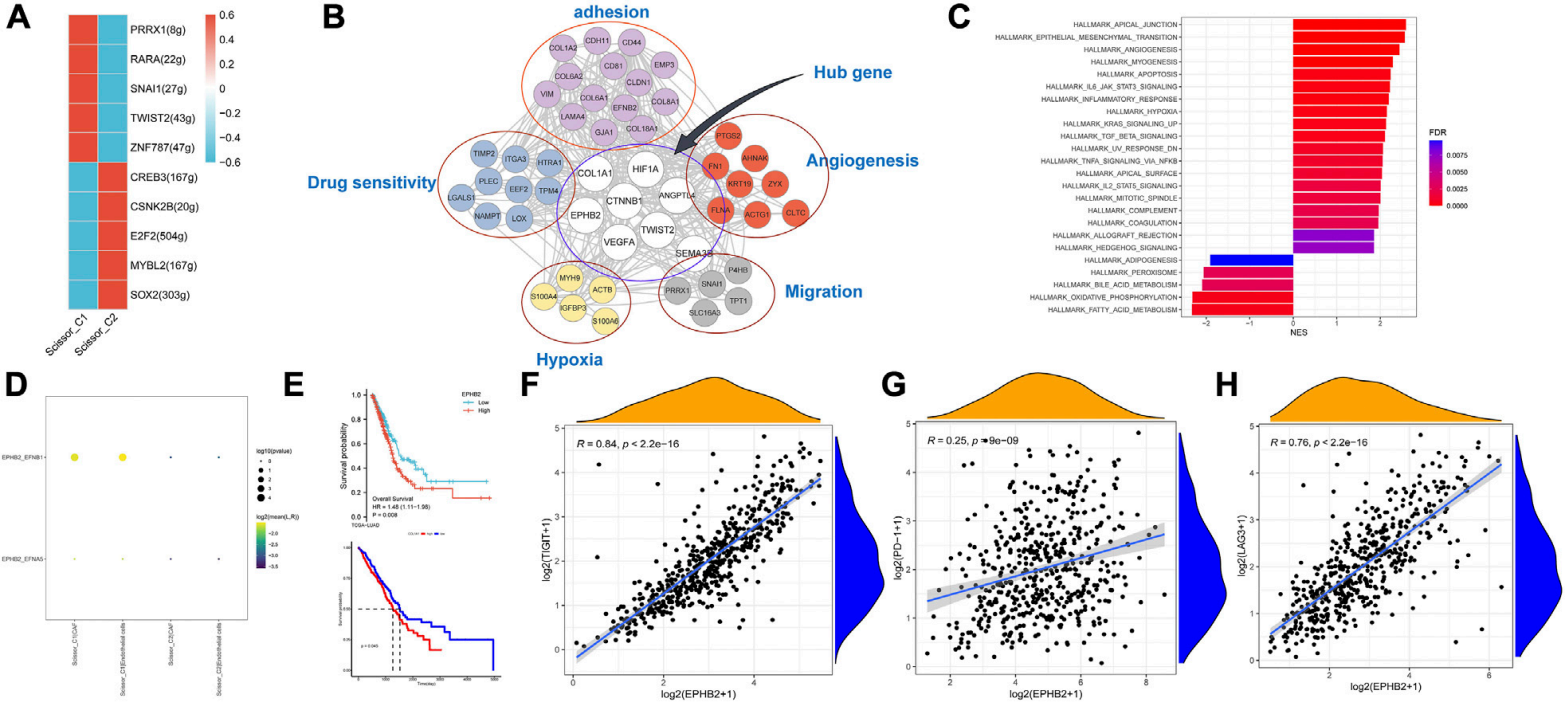
**(A)** Transcription factors significantly differentially activated by Scissor_C1 and Scissor_C2. **(B)** Scissor_C1-specific gene regulatory network. **(C)** GSEA analysis of high and low expression of COL1A1. **(D)** Scissor_C1 is specifically activated by ligand pairs with EPHB2-EFNB1 in endothelial cells. **(E)** Prognostic comparison of COL1A1 and EPHB2. **(F–H)** EPHB2 is significantly and positively correlated with immunosuppressive markers PD-1,LAG3,TIGIT.

**FIGURE 12:**
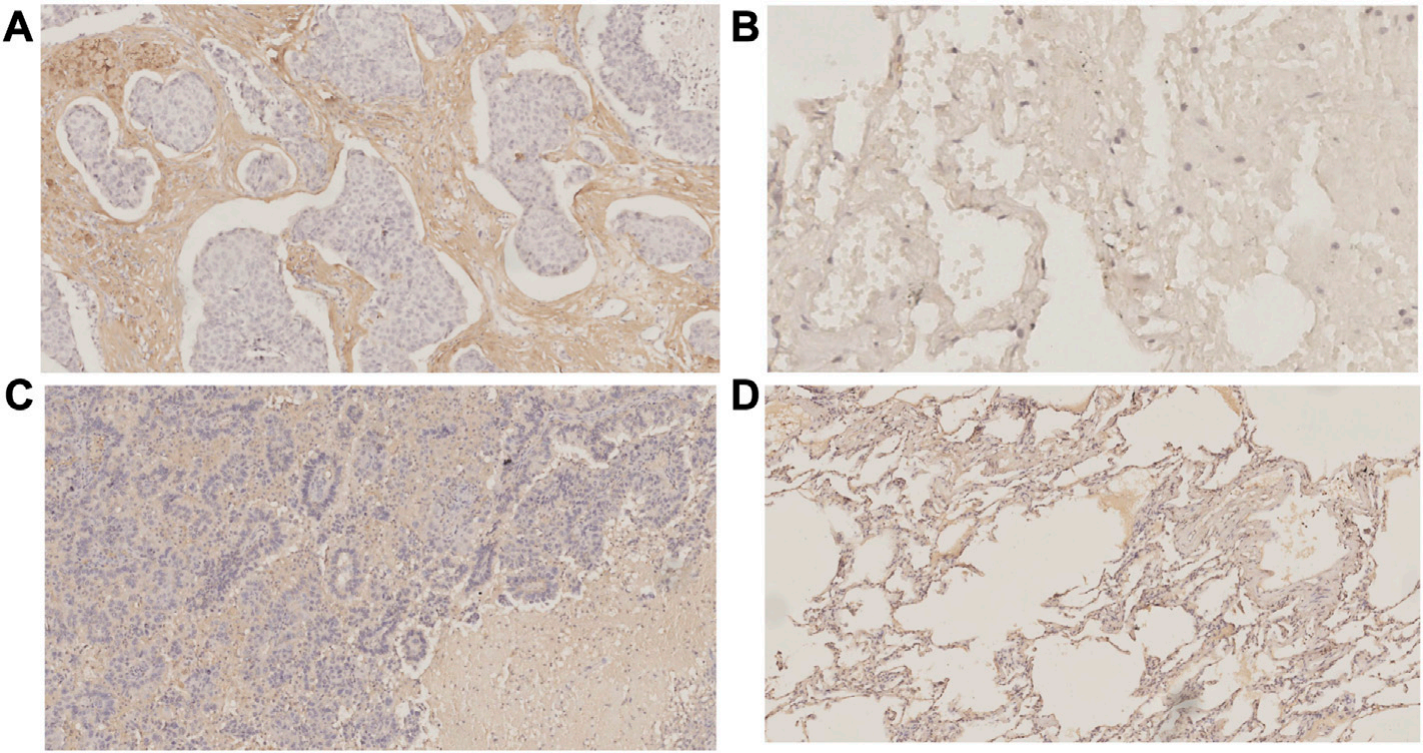
Immunohistochemistry **(A)**COL1A1 expressed in tumor tissues **(B)** COL1A1 expressed in normal tissues **(C)**EPHB2 expressed in tumor tissues **(D)** EPHB2 expressed in normal tissues.

Using pySCENIC, the top five differentially activated transcription factors between Scissor_C1 and Scissor_C2 were identified. Notably, HIF-related TWIST2 and SNAI1 transcription factors were significantly activated in Scissor_C1 (Figure 11A). Thereafter, a gene regulatory network was constructed with differentially expressed genes and top five significantly activated transcription factors of Scissor_C1. First, a protein interaction network was generated that was imported into Cytoscape 3.8.2 and then MCC centrality scores of genes were calculated using CytoHubba. The gene regulatory network was constructed from the top 50 genes with the highest centrality scores (Figure 11B). The results indicated that central regulatory network genes (*ANGPTL4, CTNNB1*, *COL1A1*, *VEGFA*, *HIF1A*, *EPHB2, TWIST2* and *SEMA3B*) were mainly associated with angiogenesis, EMT, focal adhesion, and hypoxia HIF-pathway (Figure 11B). In particular, HIF activates tumor cells to produce large amounts of β-catenin to prevent T-cell infiltration into tissues, thereby diminishing immunotherapy efficacy (Yang et al., 2016). These findings further demonstrate the invasive and metastatic nature of Sissor_C1. According to CellphoneDB, EPHB2 plays an important role in communication between Scissor_C1 and endothelial cells through EPHB2/EFNB1 axis (Figure 11D). HIF and EPHB2 are closely associated in the gene regulatory network, hypoxia induces upregulation of EPHB2 expression and affects the invasive metastatic potential of tumors, whereas EPHB2 carried by tumor cell vesicles induces angiogenesis around tumors by activating the ephrin signaling pathway (Bodin et al., 2021). At the same time, there was a significant positive correlation between EPHB2 and immune exhaustion marker (Figures 11F–H). Glutamine metabolism is significantly activated in tumor cells in hypoxic microenvironment, whereas EPHB2 is significantly associated with glutamine metabolism (Liu et al., 2022a). *COL1A1* had the highest score among key genes and was most associated with EMT and focal adhesion (Figure 11C); therefore, we chose to validate it in our own cohort sample, with baseline information shown in Supplementary Table S3. To verify COL1A1 and EPHB2 expression in LUAD, 42 patients’ tissue samples were analyzed in our clinical cohort. Immunohistochemistry results indicated that COL1A1 was highly expressed in tumor tissues, but more abundantly expressed in the stromal (Figures 12A, B), whereas EPHB2 was also highly expressed in tumor tissues (Figures 12C, D).

### 3.8 Observation of a cell-type-specific metabolic program, especially with macrophages

**FIGURE 13:**
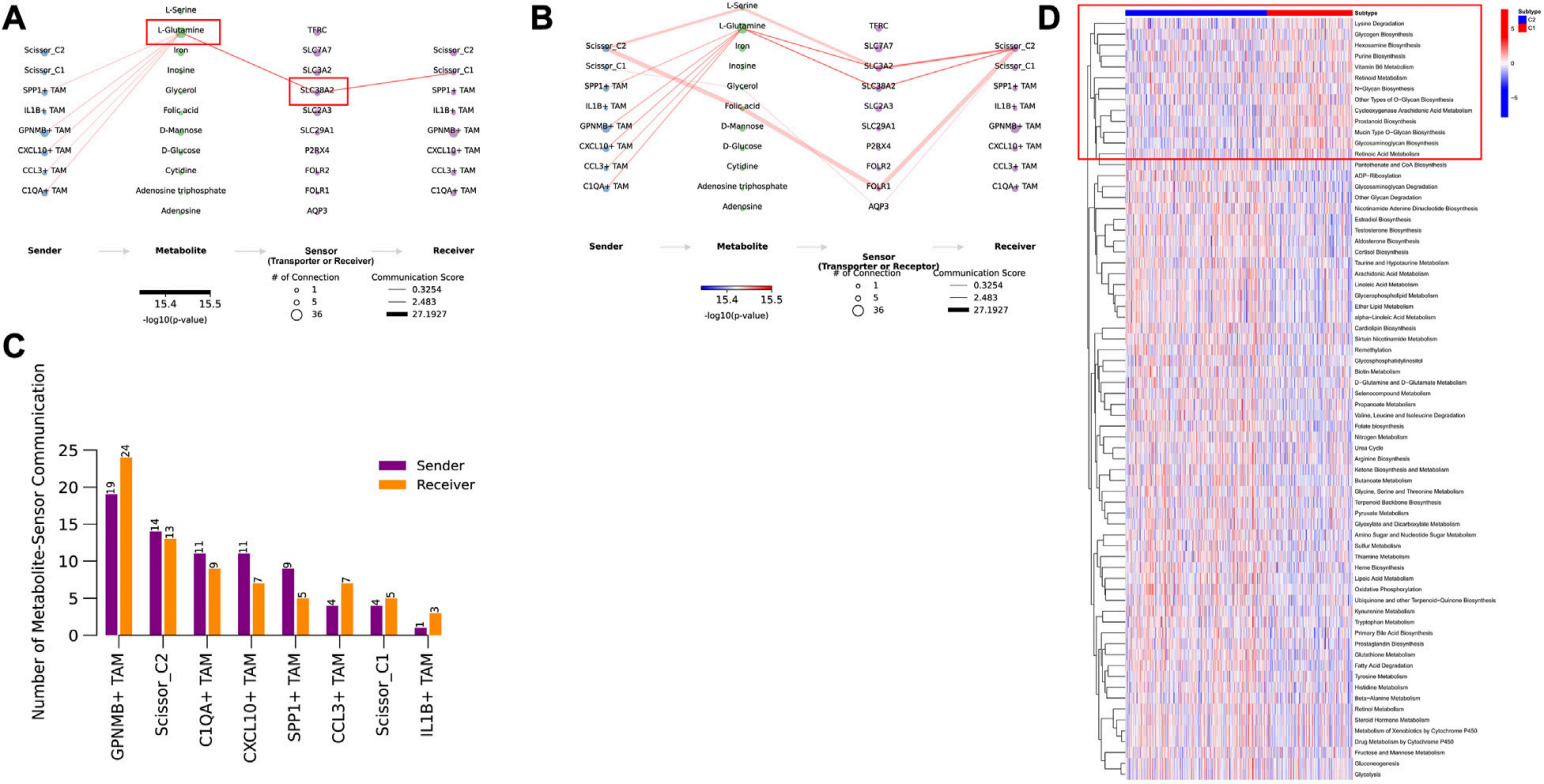
**(A)** TAM cells secreting glutamine metabolites bind to the SLC38A2 transporter protein on Scissor_C1. **(B)** TAM cells secreting glutamine metabolites bind to SLC3A2 and SLC38A2 transporter proteins on Scissor_C2 cells. **(C)** Scissor_C2 is more active in metabolite-based communication with TAM compared to Scissor_C1. **(D)** Both C1 and C2 subtypes show significant differences in metabolic pathway activation, with C2 subtype showing significant metabolic pathway activation.

Since tumor cells in the hypoxic microenvironment are metabolically reprogrammed and exhibit metabolic heterogeneity, we assessed the activity of metabolic pathways in Scissor cells using the scMetabolism method. MEBOCOST evaluation of metabolite-based intercellular communication between Scissor cells and TAM revealed that Scissor_C1 exhibits lower metabolic activity that is likely associated with a hypoxic microenvironment (Figures 13C, D). Scissor _C1 was active in the glycosphingolipid-related metabolic pathway (Supplementary Figure S2). Growth factors (IGF and TGFβ) released in hypoxic environments and adhesion receptors (e.g., integrin family) are mainly cis-interacting glycosphingolipids on the cell surface. Active glycosphingolipid metabolic pathways may lead to enhanced malignancy of tumor cells, promoting invasion and metastasis. In contrast, Scissor_C2 had higher metabolic activity (Figure 13D) and sensitivity to interactions with TAM (Figure 8C). This elevated metabolic activity may explain the positive prognosis of Scissor_C2. In the communication flow, Scissor_C1 uses the SLC38A2 transporter to uptake TAM-secreted glutamine (Figures 13A, B). Glutamine uptake is indispensable for tumor cell growth and metastasis; in LUAD, SLC38A2 plays a key role in driving glutamine endocytosis and causing glutamine addiction (Figures 13A, B). A study found that downregulation of glutamine metabolism significantly inhibited EPHB2 expression, whereas knockdown of EPHB2 significantly downregulated the expression of glutamine-related metabolic genes (Liu et al., 2022a), and glutamine metabolism was significantly correlated with EPHB2 expression. The hypoxic microenvironment possibly allows tumor cells to compete with immune cells for glutamine (Xia et al., 2021), and the significant activation of glutamine metabolism in tumor cells and the upregulation of EPHB2 expression make LUAD invasive and immunosuppressive.

### 3.9 Mechanism of invasive metastasis and immunosuppressive function of Scissor_C1

**FIGURE 14:**
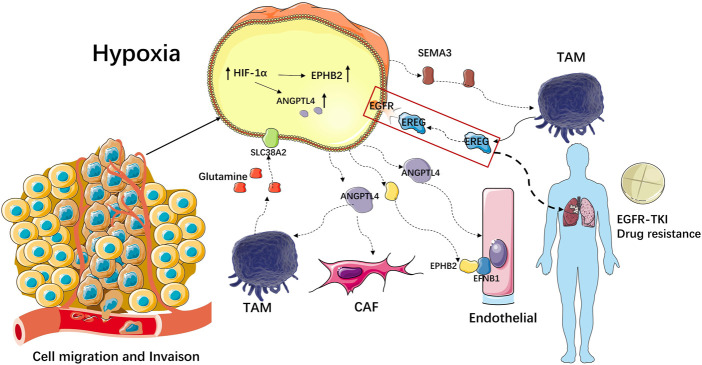
Mechanism of invasive metastasis and immunosuppressive effect of Scissor_C1.

The hypoxic microenvironment of Scissor_C1 induced high ANGPTL4 expression that then enhanced vascular permeability in endothelial cells (Niu et al., 2020), resulting in greater likelihood of tumor invasion and metastasis (Figure 14). EPHB2 expression is upregulated by hypoxia-inducible factor (HIF-1) in tumor cells like Scissor_C1, which secretes EPHB2 exotically. Interaction with EFNB1 then promoted angiogenesis in endothelial cells, as well as tumor proliferation and migration. Activation of the Wnt/β-catenin pathway prompted tumor cells to upregulate β-catenin, which in turn prevented T-cell infiltration into tumor tissue and caused immunosuppression. The binding of Scissor_C1 exocytosis SEMA3 to the receptor PLXND1 and NRP1/NRP2 on TAM may contribute to the polarization of macrophages into M2-like macrophages and thus exert immunosuppressive effects. In patients with NSCLC, TAM-secreted EREG bound to EGFR on Scissor_C1, promoting TKI resistance (Figure 14). Furthermore, TAM produced glutamine, which bound to the SLC38A2 transporter protein of Scissor_C1 and drove glutamine addiction (Morotti et al., 2021) in LUAD (Figure 14).

## 4 Discussion

In this study, we categorized TCGA-LUAD patients into two subtypes using immune microenvironment signature genes and predicted their immunotherapy response scores. Our results indicated that C1 subtypes are less likely to benefit from immunotherapy than C2 subtypes. The C2 subtype exhibits a “hot” tumor phenotype (Zhang et al., 2022a), with high infiltration of T-cell and M1-like macrophages, whereas the C1 subtype exhibits a “cold” phenotype with hypoxic and EMT features, along with decreased infiltration of immune cells (Erin et al., 2020). We then successfully used the Scissors algorithm to identify epithelial cells most associated with the two immune subtypes, SCENIC found that TWIST2 which is a key regulation of LUAD brain metastasis and SNAI1 can induce significant activation of EMT transcription factor in Scissor_C1, and further demonstrating that Scissor_C1-induced hypoxic microenvironment is prone to invasive metastasis. Scissor_C1 epithelial cells exhibited epithelial mesenchymalization, and patients with high Scissor_C1 infiltration had poor prognosis and clinical survival. The unfavorable outcome could be explained by the observation that tumor cells prone to EMT transformation were more likely to undergo local infiltration and progression.

Scissor_C1 interacted significantly with CAF, endothelial, and myeloid cells in the tumor microenvironment, specifically through highly expressing TGFBI (Batlle and Massagué, 2019). Required for the formation of immunosuppressive Treg cells, TGFβ also inhibits cytotoxic activities in NK and CD8^+^ T-cell (Batlle and Massagué, 2019). To interact with Scissor_C1, CAF secretes more VEGFA, a process possibly associated with non-response to immunotherapy (Mao et al., 2021). Relatedly, a recent study found that transient targeting of the VEGF/VEGFR axis reversed DC maturation defects (Mo et al., 2018) and reduced VEGFA-induced expression of PD-1, TIM3, and CTLA-4 in CD8^+^ T-cell (Schmittnaegel et al., 2017). In contrast, Scissor_C2 cells were positively correlated with NK and T-cell, highly expressing HLA to bind with HLA receptors on both immune cells and activating their cytotoxicity. In support of this observation, immunotherapy efficacy and survival tend to be higher in patients with metastatic melanoma who overexpress HLA (Jhunjhunwala et al., 2021). During immunotherapy, expressing HLA class II activates CD4^+^ T-cell, which help to initiate CD8^+^ T-cell and mount a successful anti-tumor immune response (Marcu et al., 2021).

Through cell communication analysis, we identified two key signaling pathways that Scissor cells uses to interact with immune and stromal cells, ANGPTL and SEMA3 signaling pathways. Activation of ANGPTL pathway mainly promotes angiogenesis and tumor metastasis in tumor regions, whereas SEMA3 pathway is mainly associated with TAM exerting immunosuppression. Scissor_C1 is highly enriched in angiogenesis, hypoxic, and EMT-related pathways. The hypoxic microenvironment induces these cells to secrete ANGPTL4. The resultant interaction of ANGPTL4 with endothelial cells decreases the latter’s connectivity while enhancing vascular permeability, allowing tumor cells to metastasize across blood vessels (Sodhi et al., 2019; Wu et al., 2021; Zhang et al., 2022b). Scissor_C1 also secretes SEMA3C, which interacts with the NRP1/NRP2 receptor of TAMs, inducing M2 polarization of macrophages and immunosuppression (Yang et al., 2022). High SEMA3C expression also promotes tumor cell angiogenesis and invasion (Yin et al., 2021). In NSCLC, EREG reduces TKI-induced apoptosis through the EGFR/ERBB2 and AKT signaling pathways (Cheng et al., 2021b), thereby contributing to TKI resistance in tumor cells. Likewise, in this study, TAM interacted with Scissor_C1 cells through the EREG-EGFR pathway, potentially contributing to EGFR-TKI (e.g., erlotinib) resistance (Ma et al., 2021).

To detect the homotypic and heterotopic characters in CAF, we used Scissor to analyze the CAF in the scRNA-seq dataset and found that CAF cells exhibited significant heterogeneity in two clusters, but homogeneity with single cancer cells clusters. We identified a subgroup of CAF caf_C1_scissor cells with immunosuppressive functions and pro-tumor metastasis. CXCL12, TGFBI, and COL3A1 expression was upregulated in the subpopulation of caf_C1_scissor cells, converging to an activated CAF cell (McAndrews et al., 2022). The secretion of CXCL12 by caf_C1_scissor induces immunosuppressive cell infiltration, CXCL12 attracts anti-inflammatory M2-like macrophage infiltration, and CXCL12 promotes tumor cell proliferation angiogenesis and metastasis (Mao et al., 2021). Binding of CAF cells secreting IGF2 to the C1QA + TAM receptor IGF2R may confer an M2-like anti-inflammatory phenotype to macrophages (Wang et al., 2020), and a study found that IGF2R-targeted activation causes proton redistribution in lysosomes and mitochondria and determines a key role in the anti-inflammatory properties of macrophages (Du et al., 2019).

Scenic transcriptional regulation analysis was used to identify significantly activated transcription factors TWIST2 and SNAI1 in Scissor_C1. TWIST2 and SNAI1 are key regulators of tumor epithelial mesenchymalization. TGFβ induces the expression of SNAI1 and TWIST transcripts, and EMT-related TFs such as TWIST and SNAI1 in turn upregulate expression of TGFβ ligands. Transcriptome sequencing revealed that EPHB2 was important in the communication between tumor and endothelial cells. HIF-1 regulates EPHB2 (Jhunjhunwala et al., 2021; Marcu et al., 2021) and activates the TGFβ pathway induced tumor cells to prevent T-cell from infiltrating into tumor tissues (Sodhi et al., 2019). The outcome was weakening of immunotherapy efficacy. In the LUAD microenvironment, EPHB2 was positively correlated with immunosuppressive molecules, such as PD-1 and LAG3. Thus, EPHB2 may play an immunosuppressive role in LUAD and promote peritumoral angiogenesis. However, the exact mechanisms underlying this role and the effects on immunotherapy require further research.

Although exhibiting very low metabolic activity, Scissor_C1 was active in glycosphingolipid-related metabolic pathways. Growth factor and adhesion receptors (e.g., integrin receptors) are mainly defined as cis-interacting molecules with cell-surface sphingolipids that mediate cellular signaling changes (Schömel et al., 2020). In melanoma, growth factor receptor-mediated activation signals are linked to adhesion signals *via* integrins, significantly upregulating downstream molecules such as AKT and pilein (Cumin et al., 2021). Finally, we used MEBOCOST to fill the technical gap of scRNA-seq-based metabolite communication, and this study introduces a further breakthrough in the study of metabolic mechanisms by using scRNA-seq to analyze cellular communication of metabolites compared to other studies. These processes heighten malignancy and increases patient susceptibility to metastasis. The high expression of SLC38A2 transporter in Scissor_C1 decreased glutamine in TAM and weakened the anti-tumor effect of immune cells. Moreover, the SLC38A2 transporter promoted glutamine addiction in tumor cells (Liu et al., 2022b), which require large amounts of glutamine for rapid growth (Huang et al., 2013). Tumor cells compete with immune cells for nutrients under hypoxic conditions, and glutamine plays an important role in immune cells, and the higher the glutamine utilization rate of immune cells, the lower the apoptosis rate. Scissor_C1 may play an immunosuppressive function by competing with TAM for glutamine through the glutamine transporter protein SLC38A2. Meanwhile, the study reports that under hypoxic environment, HIF could express EPHNB2 to uptake glutamine in cancer cells, which also enhance the glutamine accumulation.

In conclusion, this study introduced a number of advanced techniques to assess the mechanisms by which the two immune subtypes respond differently to immunotherapy and to investigate the mechanisms of immune resistance and tumor invasion and metastasis in “cold” tumors from the perspectives of immune microenvironment, metabolism, and transcriptional regulation. We also found COL1A1 and EPHB2 may be related to immunosuppression and validation in our cohort, which represents potential molecular target for enhance immunotherapy effectiveness.

## 5 Conclusion

Overall, this study explored the molecular mechanisms underlying the development of immunosuppression and metastatic resistance in the hypoxic microenvironment of LUAD from the perspective of metabolic reprogramming and cellular crosstalk. The hypoxic microenvironment of LUAD will cause a series of changes. Tumor cells will exocytose ANGPTL4 and SEMA3C molecules to help stromal cells achieve distant metastasis and weaken the anti-tumor immune function of immune cells, creating a microenvironment more suitable for the survival of malignant tumor cells, and also changing the function of immune cells to exocytose EREG to activate the EGFR pathway in tumor cells, making LUAD resistant to TKI drug. The hypoxic microenvironment also affects the metabolic reprogramming of tumor cells and the interaction of metabolite molecules between tumor and immune cells, with tumor cells predatorily feeding on glutamine from TAM cells to gain nutrients while weakening the anti-tumor function of macrophages. Ultimately, we determined that EPHB2 can be associated with immunosuppression and glutamine metabolism in LUAD and may serve as a potential therapeutic target for LUAD, providing guidance for clinical combination of drugs used in the treatment of LUAD.

## Data Availability

The original contributions presented in the study are included in the article/Supplementary Material, further inquiries can be directed to the corresponding author.
