# Supplementary figures and images for: An integrated bioinformatic analysis of bulk and single-cell sequencing clarifies immune microenvironment and metabolic profiles of lung adenocarcinoma to predict immunotherapy efficacy
Source: Front Cell Dev Biol. 2023 Apr 5;11:1163314. doi: 10.3389/fcell.2023.1163314 (PMC10113470; doi:10.3389/fcell.2023.1163314)

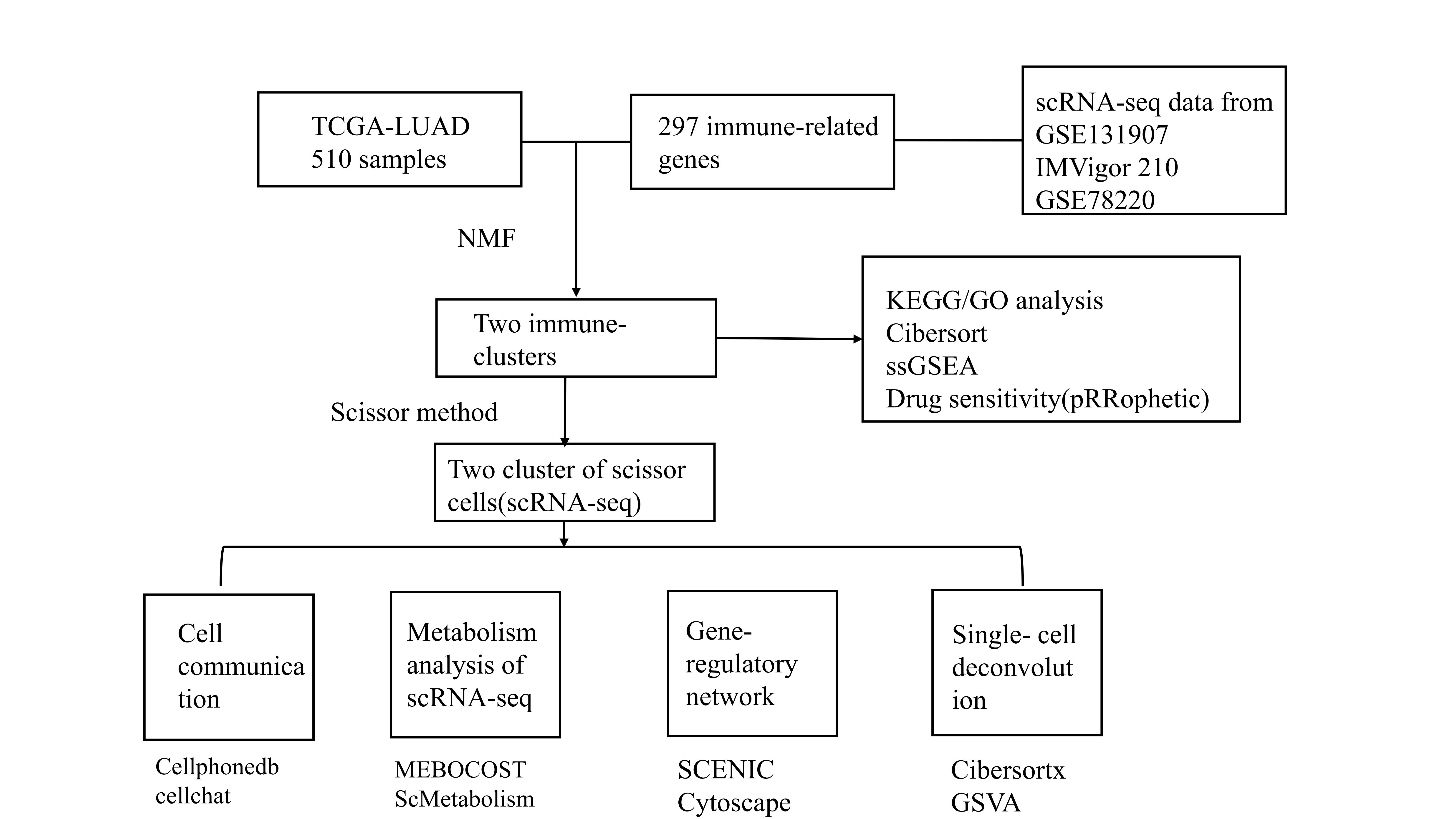

Supplement: Supplementary file 3 [file Image1.JPEG]

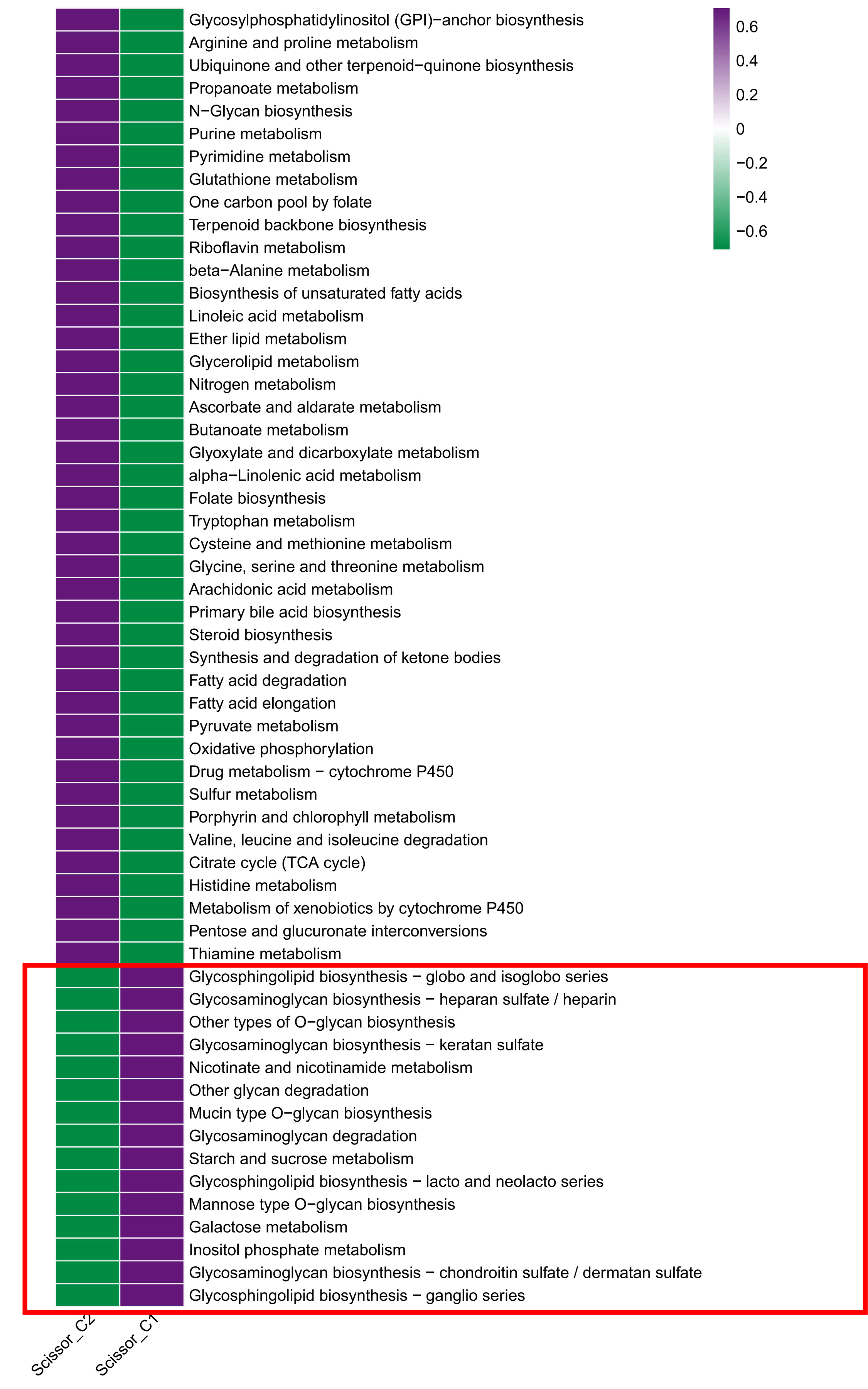

Supplement: Supplementary file 4 [file Image2.TIF]
